# The complete chloroplast genome sequence of *Davidia involucrata*

**DOI:** 10.1080/23802359.2020.1860715

**Published:** 2021-01-19

**Authors:** Li Ang, Hou Zhe

**Affiliations:** a Ecological Security and Protection Key Laboratory of Sichuan Province, Mianyang Normal University, Mianyang, China; b Key Laboratory of Southwest China Wildlife Resources Conservation (Ministry of Education), College of Life Science, China West Normal University, Nanchong, China; c MOE Key Laboratory for Biodiversity Science and Ecological Engineering, Beijing Normal University, Beijing, China

**Keywords:** *Davidia involucrata*, chloroplast genome, phylogenetic analysis

## Abstract

*Davidia involucrata Baill.* is a kind of tertiary paleotropical plant floristic relic species unique to China. This rare plant is disappearing due to poor adaptability and serious poaching. *Davidia involucrata* has been listed as a national first-level protected wild plant, a unique genus plant unique to China, a relic plant, and a well-known ornamental plant in the world. It is a national-level protected plant. In this study, the complete chloroplast genome sequence of *D. involucrata* was characterized from Illumina pair-end sequencing. The chloroplast genome of *D. involucrata* was 158,118 bp in length, containing a large single-copy (LSC) region of 87,329 bp, a small single-copy (SSC) region of 18,869 bp, and two inverted repeat (IR) regions of 25,960 bp. The overall GC content is 37.80%, while the corresponding values of the LSC, SSC, and IR regions are 36.0%, 31.6%, and 43.1%, respectively. The genome contains 132 complete genes, including 86 protein-coding genes, 38 tRNA genes, and eight rRNA genes. Phylogenetic analysis based on complete chloroplast genomes showed that *D. involucrata* and *Camptotheca acuminate* clustered together as sisters to other related species.

*Davidia involucrata* is a kind of tertiary paleotropical plant floristic relic species unique to China. *Davidia involucrata* is also named as Chinese dove tree, because the lower bud looks like a white dove ready to fly and has a high ornamental and research value. *Davidia involucrata* belongs to the family Nyssaceae. The plant, which was widely distributed during the tertiary and cretaceous periods, is now reserved only in Sichuan, Chongqing, Hubei, Guizhou, Hunan, Yunnan, and Gansu provinces of China. The dove tree is one of the key protected plants in China. But, it is still seriously threatened by human activities and overexploitation. The living fossil of this plant is gradually decreasing due to poor adaptability and serious poaching. Here, we reported the complete chloroplast genome sequences of *D. involucrata*, so as to reveal phylogenetic relationships between the species and related group.

Fresh and clean leave materials of *D. involucrata* were collected from Chengdu (102°54′E; 30°05′N) in Sichuan province, China. *D. involucrata* represents an excellent model for understanding how different evolutionary forces have sculpted the variation patterns in the genome during the process of population differentiation and ecological speciation (Neale and Antoine 2011). Meanwhile, the voucher specimens with flowers (GT001) were collected and deposited at the Herbarium of Key Laboratory of Southwest China Wildlife Resources Conservation (Ministry of Education), College of Life Science, China West Normal University. Total genomic DNA was extracted from fresh leaves by using the improved CTAB method (Doyle 1987). The obtained DNA was fragmented to construct a paired-end library with an insert-size of 350 bp, and the genome sequencing was performed using HiSeq2000 (Novogene, Tianjin, China) platform. The raw sequence data have been deposited into NCBI SRA with project accession of SRR10807901. The raw data were filtered using Trimmomatic v.0.32 with default settings . The output was a 7.0 Gb raw data of 150 bp paired-end reads. We used the software MITObim 1.8 (Hahn et al. 2013) and metaSPAdes (Nurk et al. 2017) to assemble chloroplast genomes with the cp genome of closely related species *Camptotheca acuminate* (KX904571) as the reference. Finally, we annotated the chloroplast genome using the software DOGMA (Wyman et al. 2004) with the cp genome of *Camptotheca acuminate* (KX904571) as the reference genome, and then corrected the results using Geneious 8.0.2 (Campos et al. 2016) and Sequin 15.50 (http://www.ncbi.nlm.nih.gov/Sequin/).

The annotated chloroplast genomes of *D. involucrata* were submitted to the GenBank under the accession number of MT712166. Total lengths of the chloroplast genomes were 158,118 bp. The genome had typical quadripartite structure, large single-copy (LSC) region (87,329 bp) and small single-copy (SSC) region (18,869 bp) were separated by a pair of inverted repeats (IRs) (25,960 bp), respectively. Moreover, they were composed of 132 genes, including 86 protein coding genes, 38 tRNA genes, and eight rRNA genes.

To further investigate its taxonomic status, a maximum-likelihood (ML) tree was constructed based on complete chloroplast genome sequences using MEGA 7.0 (Kumar et al. 2016) with 1000 bootstrap replicates. The program operating parameters were set as follows: a Tamura 3-parameter (T92) nucleotide substitution model with 1000 bootstrap repetitions, accompanied by Gamma distributed with Invariant site (G + I) rates, and partial deletion of gaps/missing data. We used the complete chloroplast genomes sequence of *D. involucrata* and seven other related species to construct phylogenetic tree. The eight chloroplast genome sequences were aligned with MAFFT (Katoh and Standley 2013), and then the ML tree was constructed. The phylogenetic analysis revealed that *D. involucrata* and *Camptotheca acuminate* clustered together as sisters to other related species (Figure 1).

**Figure 1.**
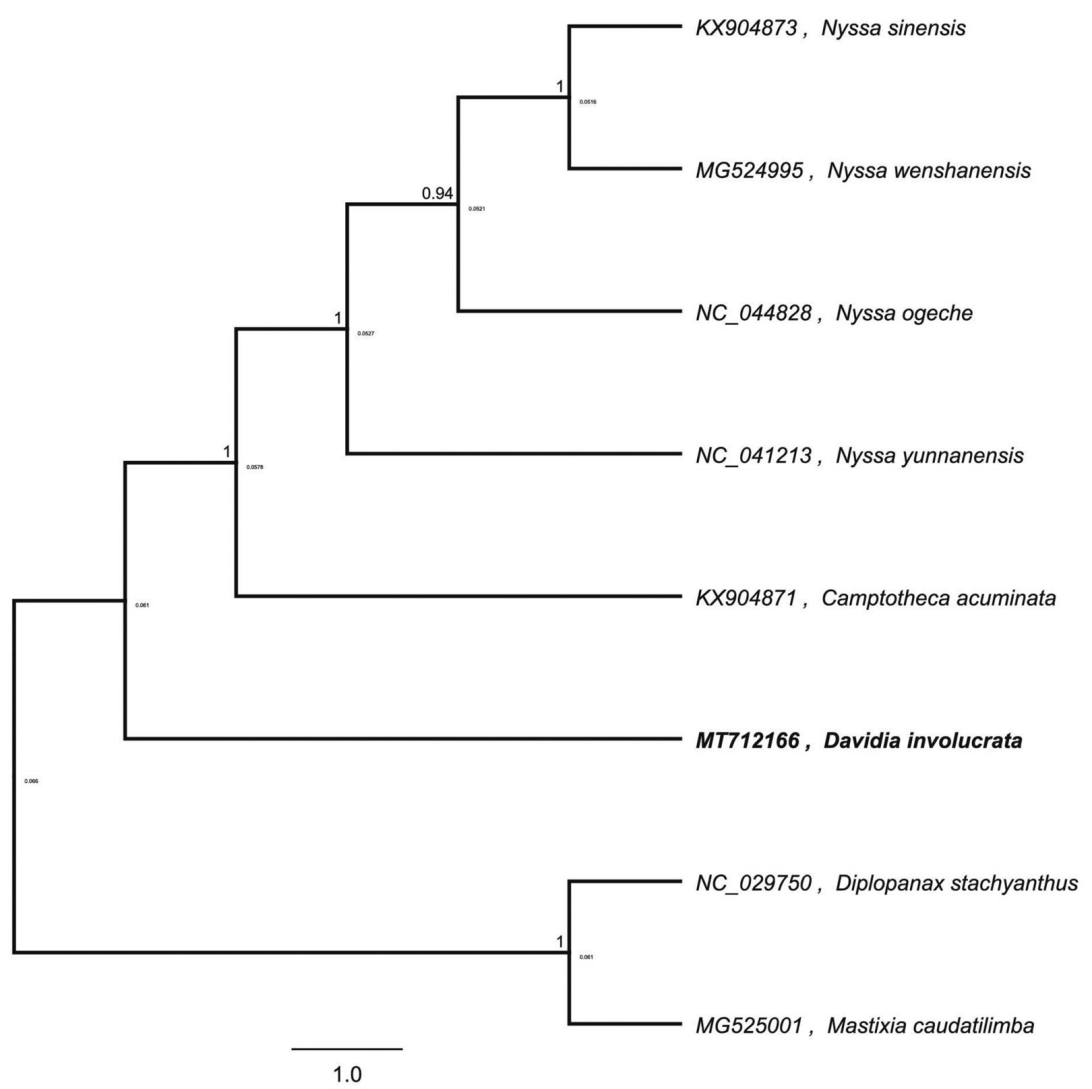
Maximum-likelihood phylogenetic tree of *Meconopsis punicea* and other related species based on the complete chloroplast genome sequence.

## Data Availability

The data that support the findings of this study are openly available in GenBank at https://www.ncbi.nlm.nih.gov, reference number MT712166.
